# Case Report: Sarcoidosis in the lymph nodes of a breast cancer patient

**DOI:** 10.12688/f1000research.20825.1

**Published:** 2019-11-05

**Authors:** Perwasha Kerio, Zain Abid, Masooma Abid, Desaar Zehra, Ghulam Haider

**Affiliations:** 1 Department of Clinical Oncology, Jinnah Postgraduate Medical Centre, Karachi, Sindh, Pakistan; 2 Jinnah Medical & Dental College, Karachi, Sindh, Pakistan

**Keywords:** breast cancer, sarcoidosis, mediastinal lymph adenopathy, positron emission tomography

## Abstract

**Background: **Sarcoidosis is an inflammatory disease that affects multiple organs in the body, especially the lungs and lymph nodes. The coexistence of sarcoidosis and breast cancer has been reported, but the coexistence of both diseases in the same patient often leads to misdiagnosis.

**Case: **We report a case of a 36-year-old woman who presented with concerns of a lump in her left breast along with pain and discharge from the nipple. On examination a 3-cm hard and tender mass was noted in the upper medial quadrant of the left breast with no palpable axillary lymph nodes.

The patient was diagnosed with an infiltrating ductal cell carcinoma of the left breast with T2N0M1 Stage IV disease, due to positive mediastinal lymphadenopathy on positron emission tomography scan. The biopsy of mediastinal lymph nodes allowed us to diagnose sarcoidosis and correctly stage her disease as T2N0M0 Stage IIA breast cancer. The patient underwent lumpectomy followed by adjuvant chemo radiotherapy and hormonal therapy - corticosteroids given for sarcoidosis up to 1 year. The patient is doing well 18 months later without recurrence of disease.

**Conclusion:** The simultaneous occurrence of both diseases in the same patient is the risk for misdiagnosis and mismanagement, therefore it is of utmost importance to correctly stage the disease with appropriate investigations and histologic confirmation prior to initiate the treatment for breast cancer.

## Introduction

Breast cancer is the most common cancer in women ^1^. It is a highly curable cancer, and with proper treatment protocols, the five-year survival of Stage IV disease is 22% ^2^. The overall survival of a breast cancer patient depends on the stage of disease with visceral or bony metastasis; therefore at the time of initial treatment planning, it is highly important to determine the intent, which is either curative or palliative. The oncological team exerts the utmost effort to properly stage cancer by giving attention to history and clinical examination for judicious use of staging workup. Usually, on examination of breast cancer patients, the axillary lymph nodes are palpable, but internal mammary lymph node involvement is less commonly seen. We present herein a rare case of breast carcinoma with sarcoidosis. Sarcoidosis is a multisystem disease that has different grades. Most patients remain asymptomatic, and very few require treatment. It is very important to differentiate and diagnose breast cancer with metastasis or breast cancer with sarcoidosis, as there is limited literature available on the coexistence of breast cancer with sarcoidosis.

## Case presentation

A 36-year-old woman presented with concerns of a lump in her left breast along with pain and discharge from the nipple. Her age of menarche was 12 years. She had no family history of breast or ovarian cancer.

We noted a 3-cm hard and tender mass in the upper medial quadrant of the left breast. There were no palpable axillary lymph nodes. The breast ultrasound showed a solid lesion with ill-defined margins in the upper medial quadrant of the left breast. The bilateral mammography demonstrated left Breast Imaging Reporting and Data System (BI-RADS) category IV and right breast BI-RADS category I.

**Figure 1.**
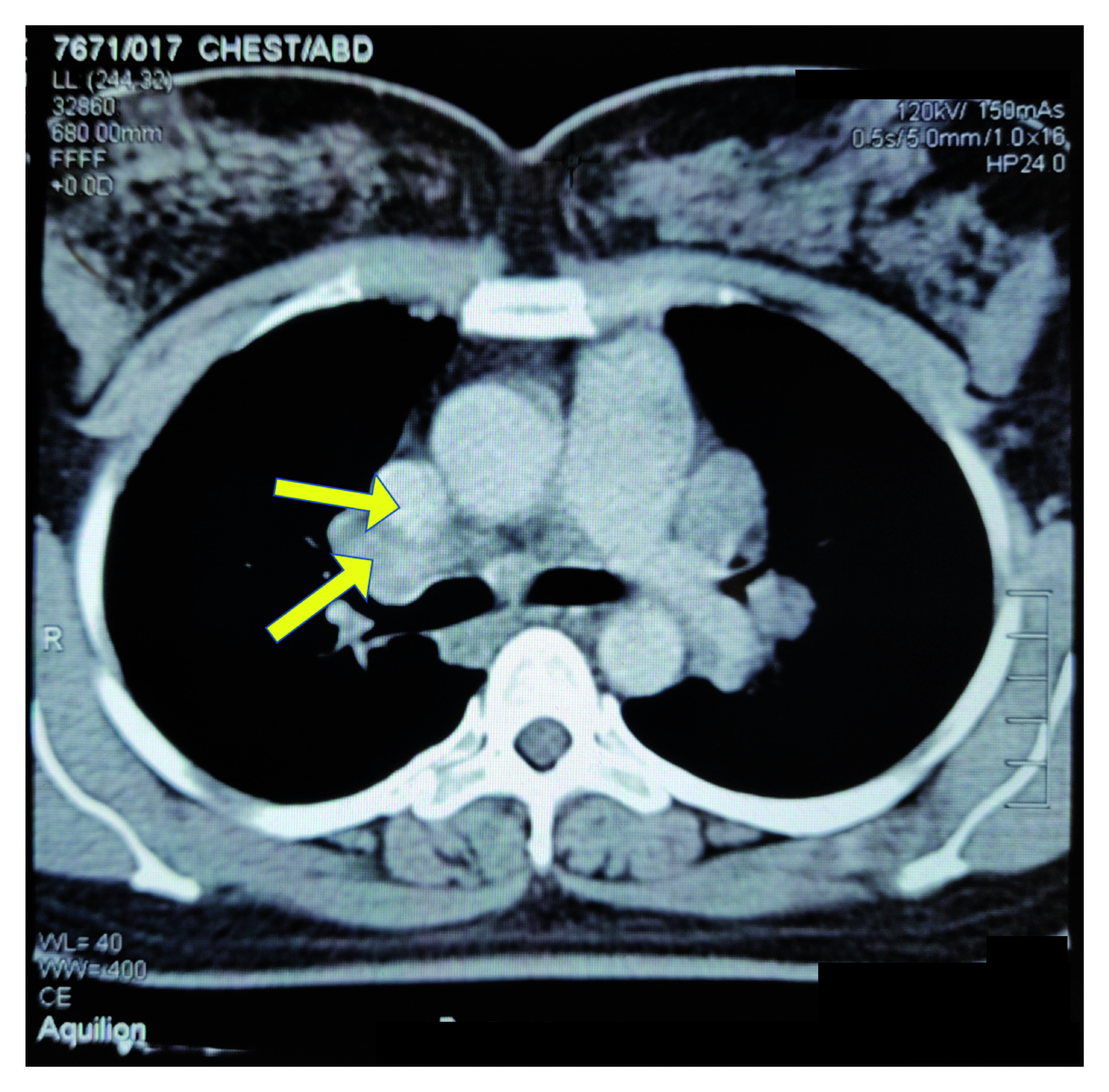
Preoperative chest computed tomography scan. Small (2.5 cm × 1.6 cm) soft tissue density mass with speculated margins in the upper quadrant of the left breast with possible focal infiltration of the underlying chest wall muscle is observed (shown by yellow arrows).

The ultrasound-guided Tru-Cut® biopsy of the mass in the left breast showed Grade 2 infiltrating ductal cell carcinoma. The preoperative chest x-ray showed bilateral hilar lymphadenopathy. The preoperative chest computed tomography (CT) scan showed a small (2.5 cm × 1.6 cm) soft tissue density mass with speculated margins in the upper quadrant of the left breast with possible focal infiltration of the underlying chest wall muscle ( Figure 1).

The results of the axillary lymphadenopathy were negative bilaterally. Multiple enlarged discrete and confluent lymph nodes were seen in the mediastinum and both hilar regions, which could have been malignant.

Positron emission tomography (PET) scan showed multiple enlarged hypermetabolic lymph nodes in the mediastinum, right paratracheal, carinal, bilateral hilar region, and aortopulmonary window ( Figure 1).

**Figure 2.**
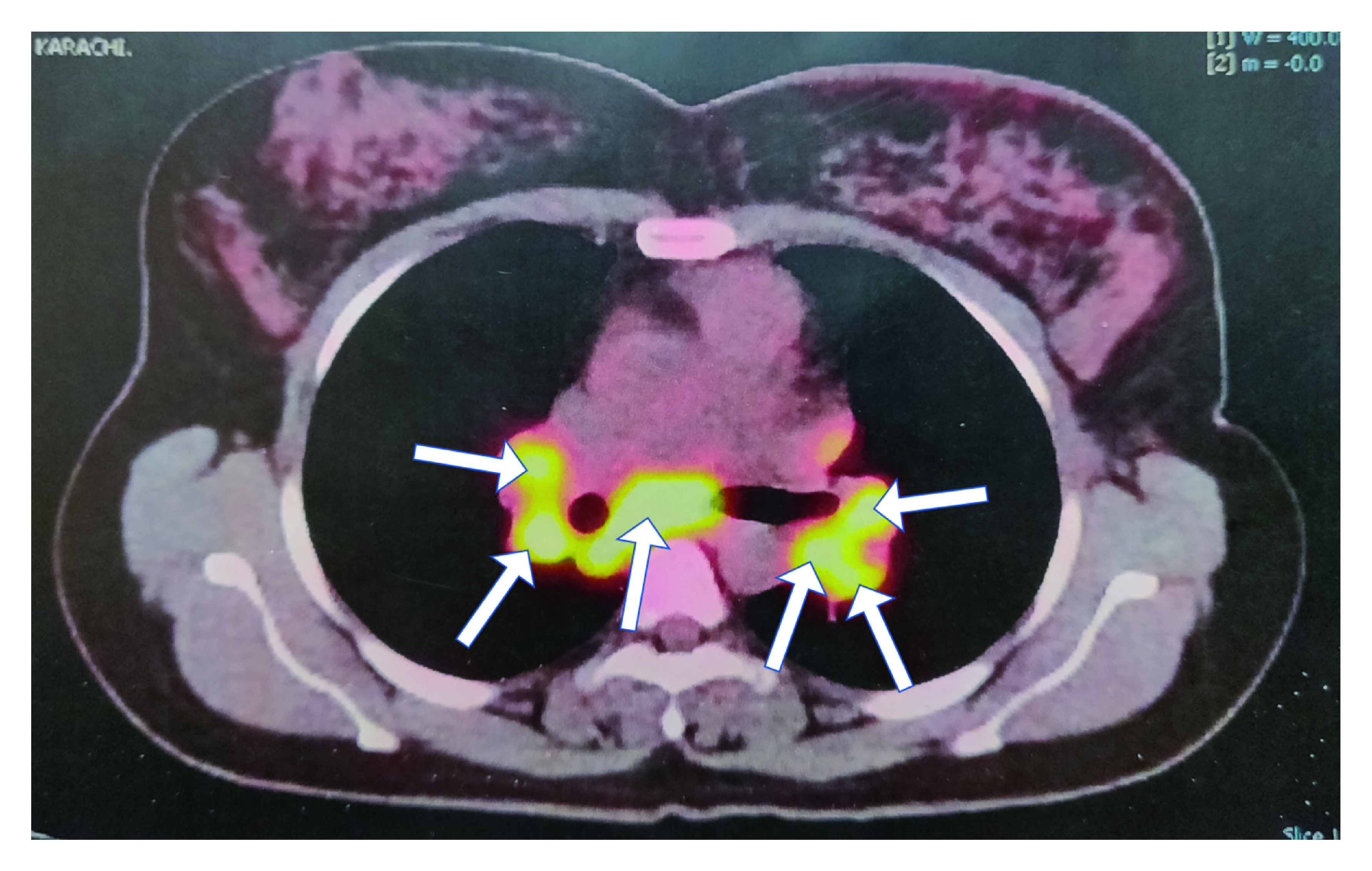
Positron emission tomography scan. Multiple enlarged hypermetabolic lymph nodes in the mediastinum, right paratracheal, carinal, bilateral hilar region, and aortopulmonary window are observed (shown by yellow arrows).

The largest lymph node in the subcranial region was 1.5 cm × 4.0 cm with a maximum standardized uptake value (SUV max) of 8.3 ( Figure 2). One of the lymph nodes in the right paratracheal region measured 2.5 cm × 2.1 cm with an SUV max of 9.9, likely representing metastasis.

The bronchoscopic biopsy of mediastinal lymph node showed non-caseous necrotic granulomatous inflammation, suggestive of sarcoidosis. This allowed us to correctly stage the disease as T2N0M0 stage IIA. The patient underwent lumpectomy followed by adjuvant chemotherapy, radiotherapy and hormonal therapy for breast cancer, while corticosteroids (prednisone, 1mg/kg for 9 months then tapered off over period of 3 months) were given for sarcoidosis for 1 year. The patient is being followed up with clinical examination every 3 months and breast mammogram done initially at 6 months following completion of radiotherapy (post mastectomy radiation therapy, total dose of 60 Gy: 50 Gy in 25 fractions given to chest wall with scar boost 10 Gy in 5 fractions) then at 1 year. At 18 months, there is no evidence of recurrence of disease and the patient is well.

## Discussion

Sarcoidosis is a multisystem granulomatous disease of unknown aetiology that manifests as non-caseating granulomas predominantly in the lungs, intrathoracic lymph nodes, and skin. The less commonly affected organs are the eyes, liver, heart, and brain. Sarcoidosis is more common in women; it occurs in patients aged 20 to 50 years ^3^. The incidence rate is higher among African Americans. Many features of sarcoidosis are suggestive of infectious origin ^4^. The diagnosis of sarcoidosis requires radiographic signs (e.g., bilateral hilar lymphadenopathy and pulmonary infiltrations) ^3^ and non-caseating granulomas on histopathology. Chronic inflammation is associated with an increased risk for malignant lymphoma and cancer in the affected tissue. The association between sarcoidosis and cancer is indistinct; although it has been proposed that patients with sarcoidosis are at increased risk for developing cancer of the lung, small intestine, stomach, liver, and skin ^4^. Cancer can produce sarcoid-like reactions in the lymph nodes, and sarcoidosis can occur in patients with cancer. The radiological features of neoplasms can be mistaken for sarcoidosis. Therefore, histologic confirmation is required before making a diagnosis ^3, 5^.

There is increased risk of breast cancer occurrence in patients with sarcoidosis than other inflammatory diseases ^5^. Lungs are common sites for breast cancer metastasis and are detected on chest radiographs in 25% of patients ^4, 5^.

Cavitation of metastatic nodule is a rare feature on radiography. However, cavitation in metastatic adenocarcinoma including breast cancer is frequently seen on CT ^5^. The axillary, mediastinal, and hilar lymph nodes are commonly involved. PET/CT imaging is superior in detecting distant metastasis in patients with stage II and stage III breast cancer, but false positive 18F-fluorodeoxyglucose (FDG) uptake and negative PET/CT are frequently seen ^6^. The conditions that cause false positive FDG uptake for malignancy include tuberculosis, fungal infection, and sarcoidosis. Initial staging with PET/CT is not recommended in preoperative early stage breast cancer because of high suboptimal sensitivity of the axillary lymph nodes, but specificity is high, and it has excellent positive predictive value confirming immediate axillary dissection instead of sentinel lymph node biopsy in the presence of high FDG uptake. However, sentinel lymph node biopsy is necessary if there is normal or slightly increased FDG uptake ^6^.

Sarcoidosis has been reported to increase levels of CA 15-3 in some cases, so the presence of this biomarker might be misleading ^7^.

## Conclusions

If abnormal FDG uptake is detected on FDG PET in isolated mediastinal lymph nodes, in the absence of axillary lymph node involvement in patients with breast cancer, a thorough preoperative evaluation is warranted with histopathologic confirmation, thereby allowing the choice of correct staging and curative strategy.

## Consent

Written informed consent was obtained from the patient for the publication of this case report and any associated images.

## Data availability

All data underlying the results are available as part of the article and no additional source data are required.
