# Supplementary material for: Case Report: Sarcoidosis in the lymph nodes of a breast cancer patient
Source: F1000Res. 2019 Nov 5;8:1853. [Version 1] doi: 10.12688/f1000research.20825.1 (PMC7968522; doi:10.12688/f1000research.20825.1)
Supplement: Peer review history

# Peer review: Reviewer response for version 1

**DOI:** 10.5256/f1000research.22905.r80201

**Published:** 2021-03-16

**Authors:** Tim L Jansen

**Affiliations:** 1 Department of Rheumatology, Expert Centre for Complex Gout, Viecuri Medical Centre, Venlo, The Netherlands

- I agree on the interest for your readership on this case report. Interesting case report on the simultaneous occurrence of breastca and granulomatous disease so called sarcoidosis.
- Diagnostic issues are elegantly depicted.
- I think the paper is ready for indexing with a minor improvement on:
  - Therapeutic considerations regarding the finding of granulomatous tissue in biopsy versus malignant cells in biopsy only.
  - Pharmacotherapeutic options.

Are enough details provided of any physical examination and diagnostic tests, treatment given and outcomes?

Yes

Is the case presented with sufficient detail to be useful for other practitioners?

Yes

Is sufficient discussion included of the importance of the findings and their relevance to future understanding of disease processes, diagnosis or treatment?

Partly

Is the background of the case’s history and progression described in sufficient detail?

Yes

Reviewer Expertise:

NA

I confirm that I have read this submission and believe that I have an appropriate level of expertise to confirm that it is of an acceptable scientific standard.

# Peer review: Reviewer response for version 1

**DOI:** 10.5256/f1000research.22905.r56717

**Published:** 2020-06-12

**Authors:** Jun Jiang

**Affiliations:** 1 National Cancer Center/National Clinical Research Center for Cancer/Cancer Hospital, Chinese Academy of Medical Sciences & Peking Union Medical College, Beijing, China

1. CT is not the first choice for breast cancer . It should provide ultrasound, X-ray and MRI imaging data.
2. Breast cancer patients without axillary lymph node metastasis, but found a large number of mediastinal swollen lymph nodes, such cases are very rare.First of all, we need to analyze the relationship between breast tumor and mediastinal enlarged lymph node. If two different diseases are considered, breast cancer is considered as breast cancer, while mediastinal enlarged lymph nodes can be metastatic lymph nodes, sarcoidosis or lymphoma.If breast and mediastinum are considered as the same  disease, lymphoma should be considered first.
3. The case report has not been discussed in detail in terms of examination method, diagnosis and differential diagnosis, and has limited value in clinical guidance.

Are enough details provided of any physical examination and diagnostic tests, treatment given and outcomes?

Partly

Is the case presented with sufficient detail to be useful for other practitioners?

Partly

Is sufficient discussion included of the importance of the findings and their relevance to future understanding of disease processes, diagnosis or treatment?

Partly

Is the background of the case’s history and progression described in sufficient detail?

Yes

Reviewer Expertise:

Diagnosis  of tumor imaging

I confirm that I have read this submission and believe that I have an appropriate level of expertise to confirm that it is of an acceptable scientific standard.
